# Antioxidative Properties of Defatted Dabai Pulp and Peel Prepared by Solid Phase Extraction 

**DOI:** 10.3390/molecules17089754

**Published:** 2012-08-14

**Authors:** Hock Eng Khoo, Azrina Azlan, Amin Ismail, Faridah Abas

**Affiliations:** 1 Department of Nutrition and Dietetics, Faculty of Medicine and Health Sciences, Universiti Putra Malaysia, UPM Serdang 43400, Selangor, Malaysia; Email: hockeng_khoo@yahoo.com (H.E.K.); amin@medic.upm.edu.my (A.I.); 2 Laboratory of Halal Science Research, Halal Products Research Institute, Universiti Putra Malaysia, UPM Serdang 43400, Selangor, Malaysia; 3 Department of Food Science, Faculty of Food Science and Technology, Universiti Putra Malaysia, UPM Serdang 43400, Selangor, Malaysia; Email: faridah@food.upm.edu.my

**Keywords:** anthocyanin, antioxidant, dabai, mass spectrometry, solid phase extraction

## Abstract

Solid phase extraction (SPE) using Sep-Pak^®^ cartridges is one of the techniques used for fractionation of antioxidant compounds in waste of dabai oil extraction (defatted dabai parts). The aim of this study was to determine the phenolic compounds and antioxidant capacity in crude extracts and several SPE fractions from methanolic extract of defatted dabai pulp and peel. Based on SPE, Sep-Pak^®^ cyanopropyl and C_18_ cartridges were used to fractionate the antioxidant-rich crude extracts into water and methanolic fractions. Analyzed using LC-MS, flavonoids, anthocyanins, saponin derivatives and other unknown antioxidative compounds were detected in the defatted dabai crude extracts and their SPE fractions. Anthocyanins were the major phenolic compounds identified in the defatted dabai peel and detected in most of the SPE fractions. Methanolic fractions of defatted dabai parts embraced higher total phenolics and antioxidant capacity than water fractions. This finding also revealed the crude extracts of defatted dabai peel have the most significant antioxidant properties compared to the methanolic and water fractions studied. The crude extract of defatted dabai parts remain as the most potent antioxidant as it contains mixture of flavonoids, anthocyanins and other potential antioxidants.

## 1. Introduction

Solid phase extraction (SPE) is one of the techniques used in separation of organic and inorganic compounds. SPE utilizing Sep-Pak^®^ cartridges has been applied for fractionation and purification of various compounds of interest. In laboratory research, SPE is commonly performed using a silica-fused column that chemically bonded. Cyanopropyl (CN), alumina, silica and C_18_ bonded-phase cartridges had been used for separation and purification of pesticides and dioxins [1,2], even phenolics and flavonoids [3,4].

Extraction and fractionation of phenolic compounds using SPE is no longer a new technique. However, fractionation of phenolic compounds by applying Sep-Pak^®^ CN and C_18_ bonded-phases are not commonly performed as CN bonded phase cartridge is typically used for separation and fractionation of chlorinated pesticides [2]. The CN silica-based polar bonded phase cartridge can be used as a less polar alternative to silica in normal-phase applications; while C_18_ is the silica-based trifunctionally-bonded octadecyl sorbent, which is also a strong and hydrophobic sorbent applies in adsorption of analytes with weak hydrophobicity from aqueous solutions [5].

Extraction and fractionation of phenolic compounds can be done in various ways using SPE. Extra care has been emphasized during sample extraction of olive oils using SPE, where it is critical to consider the type of eluent and its volume [6]. Purification of phenolic compounds from olive oil has been performed using SPE technique by injecting the oil into a C_18_ cartridge, where the methanolic fraction was collected after the cartridge had been rinsed with hexane to remove fat [7]. Sep-Pak^®^ C_18_ cartridge had been used to fractionate phenolic acids and flavonoids in grape extract after washing with water and ethyl acetate, respectively; while catechins were eluted with diethyl ether and oligomeric proanthocyanidins with methanol in the ethyl acetate fraction [8].

Dabai (*Canarium odontophyllum*) is an olive-like fruit found in the Borneo region. High total anthocyanin contents had been determined in methanol and water extracts of defatted dabai parts as compared other extraction solvents [9]. Non-defatted dabai pulp had been studied for their antioxidative properties based on *in vitro* [10] and *in vivo* [11] assays. Our preliminary study revealed that removal of fat from dabai pulp did not significantly affect the water soluble phenolic compounds in the defatted pulp. The phenolic compounds found in the defatted dabai pulp and peel can be used as nutraceutical and dietary supplement, where the defatted dabai pulp is rich in phenolics, while the purplish defatted dabai peel has high amount of anthocyanin. 

Extraction and fractionation of phenolic compounds from defatted dabai pulp and peel (as waste of dabai oil extraction) utilizing Sep-Pak^®^ CN and C_18_ bonded-phases have not been done previously. Therefore, this study aimed to extract and fractionate the phenolic compounds in the defatted dabai crude extracts based on SPE technique using Sep-Pak^®^ CN and C_18_ cartridges. This study was also performed to explore the identity of potential phenolic compounds from the defatted dabai pulp and peel and to investigate whether SPE is able to fractionate certain phenolic compounds. The water and methanol fractions obtained from SPE using Sep-Pak^®^ CN and C_18_ cartridges were determined for total phenolic content (TPC), total anthocyanin content (TAC) and antioxidant capacity. Determination of TPC using Folin-Ciocalteu reagent assay is widely known to measure crude reducing compounds, while TAC assay measures absorbance from purple color of the extracts. Although anthocyanins are part of phenolic compounds, no single assay could be able to measure all phenolics in a single plant. Major flavonoids and anthocyanins were detected using liquid chromatography-mass spectrometry (LC-MS).

## 2. Results and Discussion

Defatted dabai pulp and peel are potential antioxidant sources. The crude extracts and SPE fractions (water and methanol fractions) of defatted dabai peel and pulp-peel were studied for their total phenolic content, total anthocyanin content and antioxidant capacity based on *in vitro* assays and LC-MS detection of the potential phenolic compounds. This work is also a continuation of the study on *Canarium odontophyllum* fruit that has been performed previously by our research group. Detection of potential phenolic compounds in the defatted dabai samples was performed by matching the spectra against spectra in MS library, and also supported by previous literatures.

### 2.1. LC-MS Detection of Phenolic Compounds

**Figure 1:**
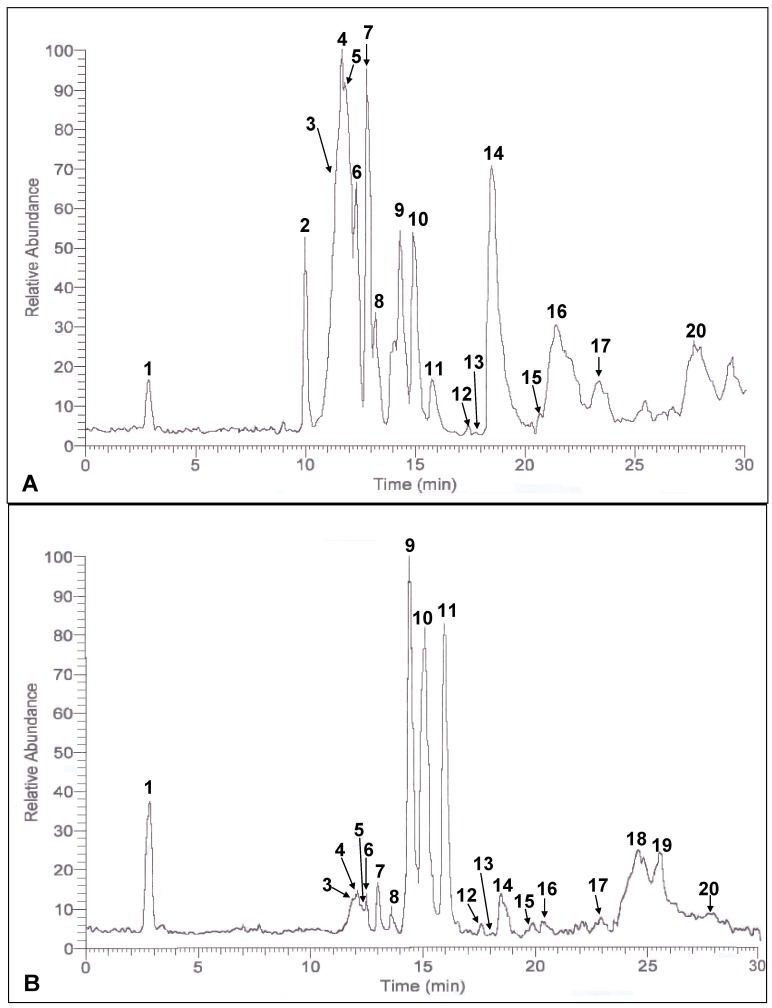
MS base peak of the defatted dabai (**A**) peel and (**B**) pulp-peel crude extracts.

Detection of potential phenolic compounds in the crude extracts of defatted dabai peel and pulp-peel, and their respective SPE fractions were based on the spectra obtained. MS base peaks of defatted dabai crude extracts are shown in Figure 1. Better ionization of phenolic compound was found for the negative ion mode. The MS/MS spectra and fragment ions obtained from some of the base peaks are not solely derived from an individual compound. It could be part of the fragment ions from one or more compounds which are trace or negligible.

The crude extract of defatted dabai parts analyzed contained a mixture of antioxidant compounds which also known to be occurred in the fresh dabai fruit [10]. Based on the data, more than 20 antioxidant compounds were detected in the crude extracts of defatted dabai peel and pulp-peel, and their SPE fractions. The relative abundance values of these compounds are shown in Table 1. The result also showed that anthocyanins were the main phenolic compounds detected in the crude extract of defatted dabai peel. Liu *et al.* [12], mentioned that anthocyanins were mainly found in the peel of purple-colored berries. Dabai peel is also purple in color. The anthocyanins detected were cyanidin, delphinidin, malvidin, hirsutidin and their glycosides. The data obtained have revealed that cyanidin was determined as major anthocyanin in the defatted dabai peel crude extract. In the studied sample, glucose and galactose were the main glycosides for cyanidin and delphinidin. However, delphinidin 3-*O*-arabinoside, cyanidin 3-*O*-arabinoside, cyanidin 3-*O*-rutinoside and cyanidin 3-*O*-sophoroside were identified in the defatted dabai peel crude extract. Besides anthocyanin, apigenin derivatives and saponin derivatives were found in the defatted dabai parts.

**Figure 2:**
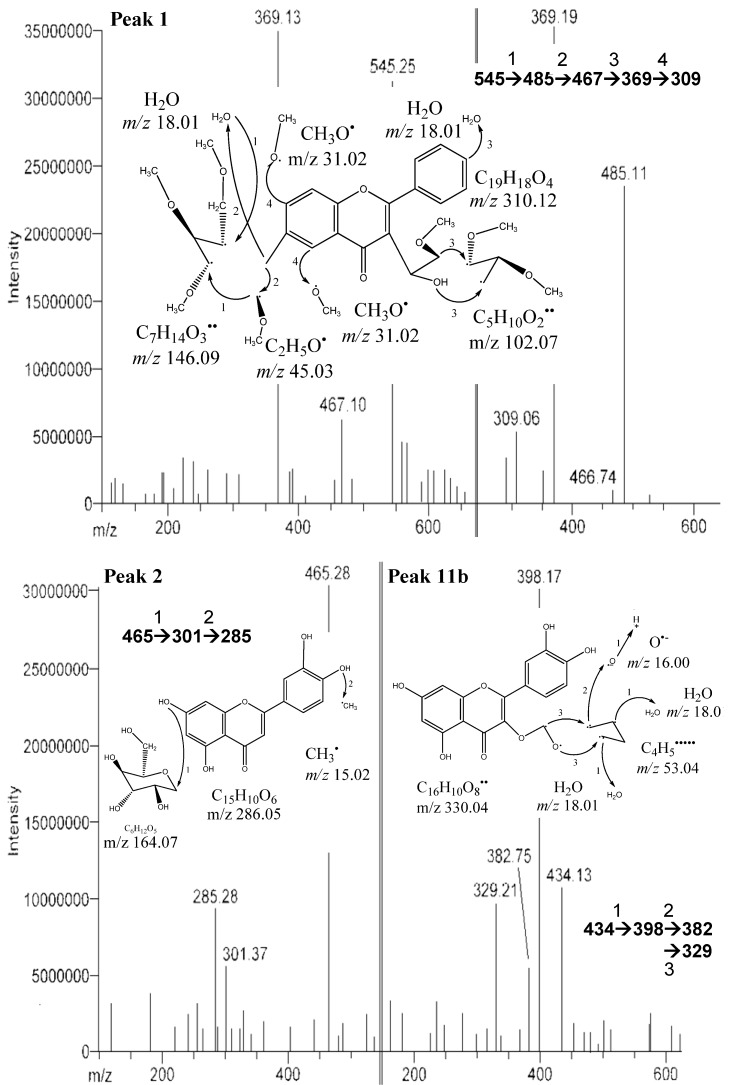
MS/MS of peaks 1 (2-*O*-β-D-xylosyl-8-C-β-D-galactosylapigenin, 2 (hesperetin 7-*O*-glucoside) and 11b (quercetin 3-*O*-α-D-arabinopyranoside) from the defatted dabai crude extract.

Among the peaks detected, peak 1 was identified as an apigenin derivative (Figure 2). This is in agreement with a study reported by Bakhtiar *et al.* [13], that the compound (permethyl derivative of 2-*O*-β-D-xylosyl-8-*C*-β-D-galactosylapigenin) has similar fragment ions at *m/z* 630, 545, 515, 449, 485, 467, 397, 371, 369, 367, 355, 341, 339, 325, 323, 312, 311 and 309 as peak 1. However, based on the fragment ions from the identified peak, *m/z* 485, 467, 369, 309 (Table 2) were spotted. It shows that complete ionization of the compound had not occurred. Peak 2 was identified as flavonoid glucoside ([M−H]^−^ at *m/z* 465 and the fragment ions at *m/z* 301, 285) (Figure 2). Similar fragment ions have been found in pure compound isolated from the leaves of *Cerbera manghas* as reported by Sakushima * et al.* [14], where the identified compound was hesperetin 7-*O*-glucoside. Besides, three anthocyanin derivatives were spotted in the defatted dabai peel crude extract. These derivatives have [M−2H]^−^ at *m/z* 479, 485 and 487, and their molecular structures are unknown. Peak 3 (shoulder peak) was determined as a mixture of cyanidin 3-*O*-sophoroside and cyanidin 3-*O*-rutinoside based on the *m/z* values found, while peaks 4 and 5 were cyanidin 3-*O*-glucoside and cyanidin 3-*O*-galactoside, respectively. Besides, cyanidin 3-*O*-arabinoside ([M−2H]^−^ at *m/z* 419) and hirsutidin 3-*O*-glucoside ([M−2H]^−^ at *m/z* 505) were found to co-exist in peak 5. Moreover, malvidin 3-*O*-glucoside ([M−2H]^−^ at *m/z* 491) was co-identified with glucosides of apigenin from peaks 6 and 7.

**Table 1:** Values of the relative abundance ^a^ from the MS base peaks for the crude extracts of defatted dabai parts and their SPE fractions.

Peak	Peel							Pulp-Peel					
	Extract	CN-H_2_O	CN-MeOH	C_18_-H_2_O	C_18_-MeOH	Residual		Extract	CN-H_2_O	CN-MeOH	C_18_-H_2_O	C_18_-MeOH	Residual
1	17.7	3.5	–	7.7	–	7.0		37.3	29.6	14.5	13.4	–	38.9
2	**50.0**	6.6	16.0	26.0	4.7	6.6		–	–	–	–	–	–
3	79.1	82.1	33.5	60.5	10.1	11.2		9.7	12.2	10.7	5.9	1.1	24.3
4	100	100	38.5	61.2	16.4	12.7		11.6	18.2	12.2	7.8	2.1	29.0
5	92.7	85.1	33.0	63.8	14.5	11.2		8.1	18.0	9.8	5.1	1.5	27.7
6	64.1	75.1	30.2	30.0	13.9	–		11.6	18.3	24.0	16.0	6.6	16.9
7	90.1	21.3	42.9	30.6	21.7	18.1		17.3	20.1	35.5	18.2	7.9	6.4
8	37.3	24.1	33.9	25.6	27.8	4.3		7.8	15.6	39.7	23.9	18.2	5.5
9	57.6	71.7	39.5	85.3	6.9	100		100	100	87.9	100	10.0	100
10	58.8	82.5	37.6	84.3	7.4	78.1		81.1	88.1	90.0	95.9	10.0	77.6
11	19.5	25.3	100	100	100	30.9		87.3	22.0	100	51.6	100	32.8
12	3.69	16.2	16.1	8.5	13.0	3.2		2.7	–	5.8	10.5	14.1	–
13	3.66	21.0	36.9	16.1	15.6	5.1		9.38	–	10.8	20.5	23.3	35.8
14	71.7	20.2	46.2	16.1	19.5	12.6		15.1	–	60.9	23.7	33.6	36.0
15	3.71	33.2	42.7	27.3	31.8	20.5		2.6	–	9.5	13.0	30.6	21.7
16	33.5	9.6	9.1	31.1	7.5	29.5		5.5	80.2	–	59.1	–	8.2
17	18.8	34.5	37.7	20.1	2.0	25.3		6.4	37.9	–	37.9	–	40.4
18	–	–	–	–	–	–		20.2	–	48.5	–	2.5	4.3
19	–	–	–	–	–	–		9.64	–	61.7	–	–	8.6
20	26.8	53.6	12.4	33.0	3.9	2.9		7.5	59.2	7.8	50.1	–	68.4

^a^ Relative abundance means the distribution of the amount of phenolics as observed in the chromatogram (in percentage).

**Table 2:** Chromatographic and spectral properties of compounds detected in defatted Dabai waste.

Peak No.	Identity	*t*_R_(min)	λ _max _(nm)	[M−H]^−^/[M−2H]^−^ ( *m/z*)	Fragment ions ( *m/z*)
1	Apigenin derivative	2.9	235, 570	545	485, 467, 369, 309
2	Hesperetin 7- *O*-glucoside	10.0	240, 520	465	301, 285
3	Cyanidin 3- *O*-rutinoside	11.4	270, 515	593	285
	Cyanidin 3- *O*-sophoroside			609	285
4	Cyanidin 3- *O*-glucoside	11.7	275, 515	447	285
5	Cyanidin 3- *O*-galactoside	11.9	270, 515	447	285
	Cyanidin 3- *O*-arabinoside			417	285
	Hirsutidin 3- *O*-glucoside			505	–
6	Apigenin 8- *C*-glucoside (Vitexin)	12.4	250, 515	431	413, 341, 311, 269
	Malvidin 3- *O*-glucoside			491	–
7	Apigenin 6- *C*-glucoside (Isovitexin)	12.8	255, 515	431	413, 353, 341, 311, 269
8	Delphinidin 3- *O*-glucoside;Delphinidin 3-*O*-galactoside	13.2	250, 520	463	301
	Delphinidin 3- *O*-arabinoside			433	301
9	Saponin derivative	14.3	250, 525	677	659, 564, 451, 338
10	Saponin derivative	14.9	260, 530	850	790, 772, 677, 659, 564
11 (a)	Methyl 4,5-dicaffeoylquinate (pulp)	15.8	270, 535	529	511, 367, 349
11 (b)	Quercetin 3- *O*-α-D-arabinopyranoside (peel)		275, 550	434	398, 382, 329
12	–	17.4	270, 555	561	525, 456
13	–	17.8	270, 555	331	271, 255, 230, 202
14	Saponin derivative	18.5	270, 550	537	815, 712, 682, 585, 537, 458, 398, 331, 329
15	–	19.6	275	772	585, 458, 403, 343, 271
16	–	21.5	270	325	–
17	–	23.4	275	325	–
18	–	25.6	275	766	753. 734, 481
19	–	26.4	275	794	780, 766, 512
20	–	27.7	275	339	311, 217, 183

Notes: (a) refers to the compound identified in the defatted dabai pulp-peel and (b) refers to the compounds identified in the defatted dabai peel.

**Figure 3:**
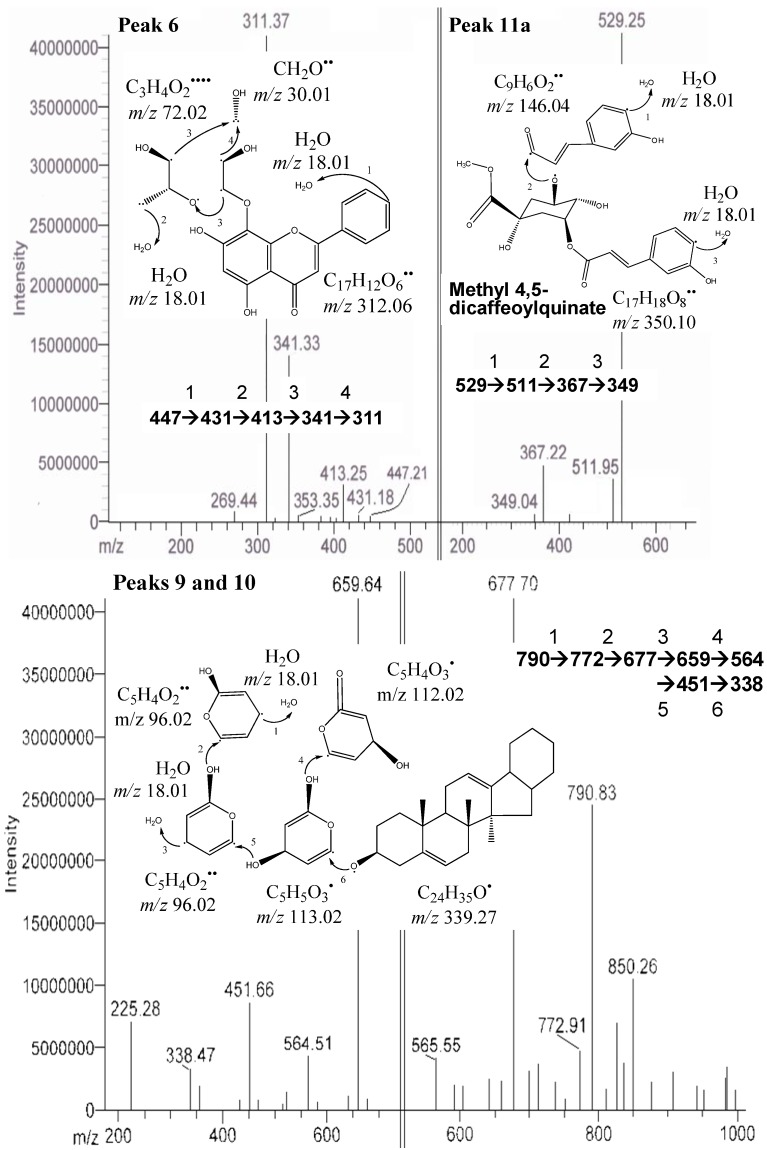
.MS/MS of peaks 6 (apigenin 8-*C*-glucoside), 9 & 10 (saponin derivatives), and 11a (methyl 4,5-dicaffeoylquinate) from the defatted dabai crude extract.

In this study, apigenin glucosides were detected in the defatted dabai sample. The amount of apigenin glucosides was high in crude extract of the peel, but lower in the pulp-peel crude extract. The identified apigenin glucosides were apigenin 8-*C*-glucoside (vitexin-peak 6) (Figure 3) and apigenin 6-*C*-glucoside (isovitexin-peak 7). Similar findings were reported by Sánchez-Rabaneda *et al.* [15], that these two glucosides of apigenin were also found in cocoa extract. The [M−H]^−^ of these compounds were at *m/z* 431 with the MS/MS ions scanned at *m/z* 341, 311, 269 (vitexin) and *m/z* 353, 341, 311, 269 (isovitexin). Besides, the negative ion fragments of the peak 8 were identified as glycosides of delphinidin. They are delphinidin 3-*O*-glucoside/galactoside (*m/z* 463), delphinidin 3-*O*-arabinoside (*m/z* 433), and *m/z* 301 for delphinidin. 

The compound from peaks 9 and 10 was predicted as one of the unknown saponin derivatives. It was postulated as a compound with huge molecular structures and has [M−H]^−^ at *m/z* 790 and the fragment ions at *m/z* 850, 772, 677, 659 and 564 (Table 2). Difference in its four fragment ions identified was at *m/z* 113 and had a smallest fragment ion at *m/z* 338 (Figure 3). It was predicted to be a saponin derivative. The assumption is supported by Li *et al.* [16], who also found a similar structure from the roots of *Pulsatilla campanella* and it was named as saponins leontosides. Besides anthocyanins, the compound identified from peaks 9 and 10 was also one of the major compounds identified in the defatted dabai sample.

Another saponin derivative was found at a retention time of 18.5 min (peak 14). It has the smallest fragment ion at *m/z* 375, [M−H]^−^ at *m/z* 537 and the other fragment ions at *m/z* 815, 712, 705, 585 (Table 2). Besides, trace amounts of vanillic acid and protocatechuic acid were detected in the defatted dabai pulp-peel crude extracts (data not shown). Based on the MS data obtained, these two phenolic acids were tentatively identified as protocatechuic acid (*m/z* 154) and vanillic acid (*m/z* 164) with retention times of 11.3 min and 9.5 min, respectively. Other phenolic acids were not found in the crude extract of defatted dabai parts. This could be due to the optimized extraction processes that involved mild heat treatment resulted in degradation of the most phenolic acids. Spanos and Wrolstad [17] also supported the facts that heat induced during food processing causes degradation of phenolic compounds.

One of the unknown major peaks (peak 11a) were identified as methyl 4,5-dicaffeoylquinate in the defatted dabai pulp, while quercetin 3-*O*-arabinopyranoside (peak 11b) was determined in the defatted dabai peel (Figure 3). Both compounds were found at similar retention time of 15.8 ± 0.2 min. The molar mass of compound from peak 11a was 530 (C_26_H_26_O_12_), with [M−H]^−^ at *m/z* 529 and the fragment ions at *m/z* 511, 367, 349 (Figure 3). The difference between *m/z* 529 and 349 is a loss caffeic acid at *m/z* 180. The molecular structure of this compound (peak 11a) is a combination of a methyl quinate and two caffeic acid. This compound (methyl 4,5-dicaffeoylquinate) has been characterized by Ma *et al.* [18], where it has *m/z* 529 and 367. The fragment ions at *m/z* 511 and 349 are part of the other fragment ions found. The compound for peak 11b has [M−H]^−^ at *m/z* 434 and the fragment ions at *m/z* 398, 382, 329 (Figure 2). Sanbongi *et al.* [19] supported our finding that same compound (quercetin 3-*O*-α-L-arabinopyranoside) was isolated from cacao liquor, which has [M+H]^+^ at *m*/*z* 435. 

**Figure 4:**
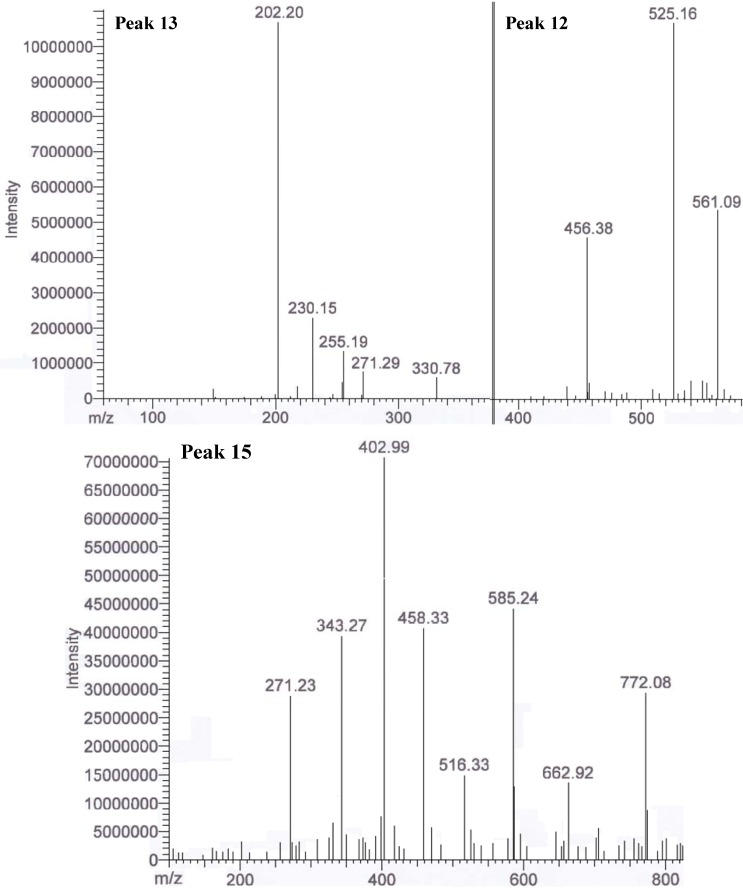
.MS/MS of peaks 12, 13 and 15 (unknown compounds) in the extract of part of a defatted dabai fruit.

From the result obtained, peaks 12 to 20 were determined as unknown compounds, except for peak 14 (saponin derivative). Some of these peaks had large molecular ions (*m/z* 600–800). Peaks 12, 13 and 15 were the potentially unknown antioxidants. These peaks were not able to identify as either phenolics or terpenes due to the fragments ions were not matched for any of the known phenolic compounds (Figure 4). One or two major peaks were also found at the end of the chromatogram. However, these peaks were not fully ionized into fragment ions. Therefore, identification of potential phytochemicals is impossible. Isolation of these compounds is recommended in future studies for NMR analysis to further characterize these few unknown major peaks that have been discovered in the crude extracts of defatted dabai fruit. Besides, more than 10 other unknown minor peaks were not identified as the amount is in trace or might not be detectable. Peak 2 was not spotted in the defatted dabai pulp-peel crude extract, but it was detected in the defatted dabai peel crude extract. Therefore we conclude that peak 2 (hesperetin 7-*O* glucoside) was not occurred in defatted dabai pulp that without the peel. Moreover, peaks 18 and 19 were not found in the defatted dabai peel. Future studies are required to identify and characterize these unknown peaks using NMR.

Fractionated using SPE, anthocyanins were detected in all SPE fractions of the defatted dabai pulp and peel crude extracts, except for cyanidin 3-*O*-arabinoside, which was not found in the CN-methanol (CN-MeOH) and C_18_-methanol (C_18_-MeOH) fractions. Cyanidin 3-*O*-rutinoside, cyanidin 3-*O*-sophoroside and hirsutidin 3-*O*-glucoside were not detected in all SPE fractions. Identification of anthocyanins in the defatted dabai parts was further confirmed based on a method described by Abad-García *et al.* [20]. Four major anthocyanin peaks were found in the defatted dabai peel, which co-exist together with three to four other minor peaks. However, anthocyanins were the only phenolic compounds that detected at 530 nm. For the major antioxidants detected as in the peaks 4, 5 and 7, the levels of these compounds were found to be higher in the defatted dabai peel fractions than the levels in defatted dabai pulp fractions that have been determined based on the relative abundance values obtained from the MS base peaks (Table 1). On the other hand, the levels of other major compounds from peaks 9 and 10 were high in both SPE fractions of defatted dabai peel and pulp-peel. Nonetheless, these compounds were found to be higher in the defatted dabai pulp than the peel. 

The results also revealed that the other non-identified (unknown) compounds (peaks 15–20) that were detected in the methanolic fractions were not detected (trace) or found to occur in low levels. The compounds identified as in peaks 9 and 10 were predicted to highly soluble in water due to the high peak intensity observed for the water fractions studied. Conversely, peak 11a (methyl 4,5-dicaffeoylquinate) was highly solubilized in methanol. The carboxyl group of methyl 4,5-dicaffeoylquinate should have enhanced its solubility in water [21]; however, this compound is highly solubilized in methanol. The reason is unknown. Quercetin 3-*O*-arabinopyranoside (peak 11b) is a semi-polar compound, which is able to solublilize in both methanol and water. Comparing the two Sep-Pak^®^ cartridges used, CN stationary phase was not able to completely retain the antioxidant compounds detected in defatted dabai parts. The possible reason is that based on the sequential design of SPE, the non-retained antioxidants collected from CN cartridges were finally retained in C_18_ cartridges. Besides, both methanol and water fractions obtained from CN and C_18_ cartridges have similar intensity for most of the peaks observed. 

### 2.2. TPC, TAC and Antioxidant Capacity

**Figure 5:**
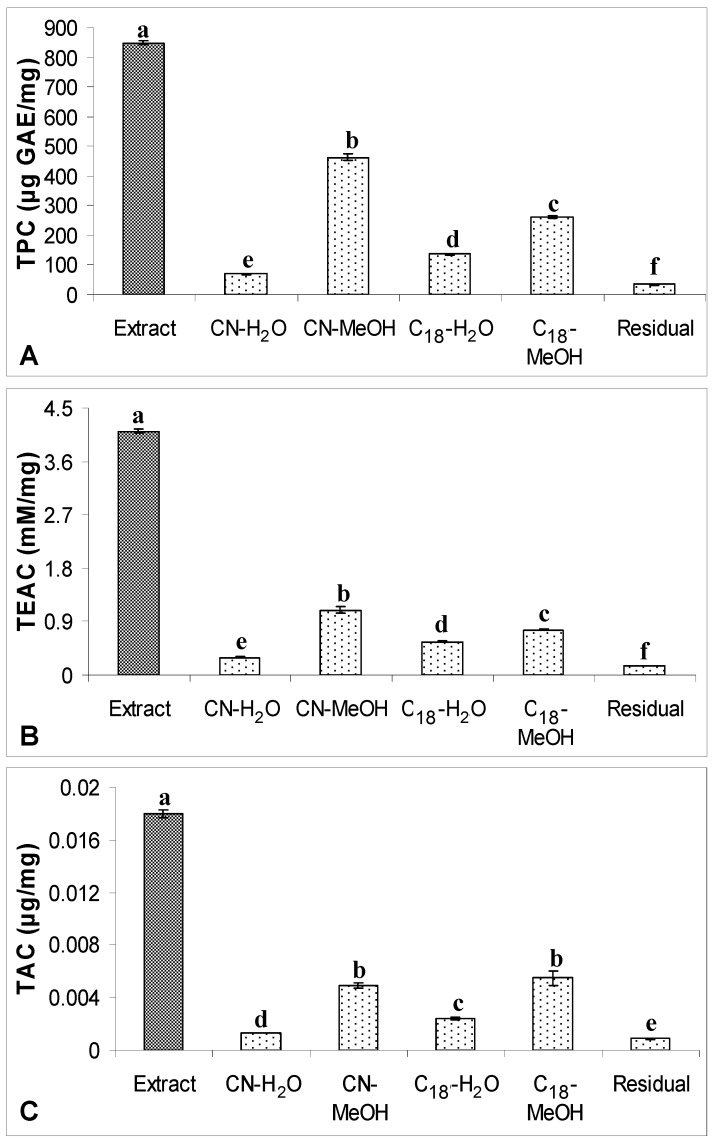
(**A**) TPC, (**B**) TEAC and (**C**) TAC of the crude extract of defatted dabai peel (5 mg/mL) and their SPE fractions. The data are presented as the mean ± SD of three replicates. Different lowercase letters (a–f) indicate a significant difference (*p* < 0.05).

**Figure 6:**
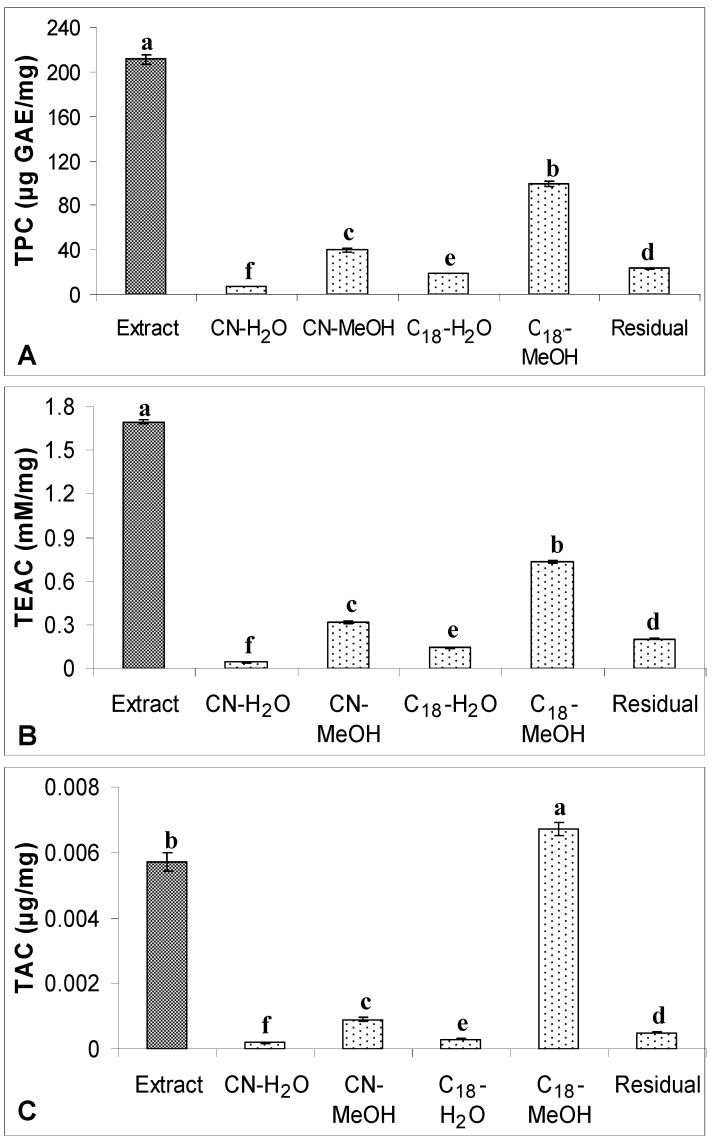
(**A**) TPC, (**B**) TEAC and (**C**) TAC of the crude extract of defatted dabai pulp-peel (5 mg/mL) and their SPE fractions. The data are presented as the mean ± SD of three replicates. Different lowercase letters (a–f) indicate a significant difference (*p* < 0.05).

TPC, TAC and antioxidant capacity of defatted dabai crude extracts and their SPE fractions (water and methanolic fractions) are shown in Figure 5 and Figure 6. LSD *post-hoc* comparison revealed that methanol and water fractions obtained from the defatted dabai peel crude extract had significantly lower levels of TPC, TAC and TEAC than the crude extract (*p* < 0.05). Among the fractions studied, both CN-MeOH and C_18_-MeOH fractions had higher levels of TPC, TAC and TEAC as compared to the water fractions. LSD *post-hoc* comparison also revealed that the antioxidant properties found in the CN-MeOH fraction of defatted dabai peel were significantly higher than the other SPE fractions, except for TAC where no significant difference was found between the CN-MeOH and C_18_-MeOH fractions. Residual fractions were mainly consisted of phytochemicals that unretained by CN and C_18_ bonded-phases. These fractions had lower levels of TPC, TAC and TEAC as compared to other SPE fractions. It shows that the Sep-Pak^®^ cartridges have successfully fractionated the crude extracts of defatted dabai peel and pulp-peel, thus recovered most of the antioxidant compounds. Similar as found for the crude extract of defatted dabai peel, LSD *post-hoc* test showed that the crude extract of defatted dabai pulp-peel had significantly higher levels of TPC and TEAC than the SPE fractions, except for TAC where C_18_-MeOH fraction had significantly higher of level of TAC than the crude extract.

In general, methanolic fraction of defatted dabai pulp-peel that was obtained from C_18_-bonded stationary phase had the highest antioxidant properties as compared to the other SPE fractions. Water fraction from CN-bonded stationary phase (CN-H_2_O) of the defatted dabai pulp-peel had the lowest levels of TPC, TAC and TEAC as compared to other SPE fractions; while the residual fraction of defatted dabai pulp-peel crude extract had significantly higher levels of antioxidant properties than the CN-H_2_O fractions. This is one of the exceptions that some antioxidants from the defatted dabai pulp were not fully recovered from Sep-Pak^®^ CN and C_18_ cartridges. It is suggested that other types of SPE cartridge should be used to fractionate and recover the potential antioxidants in different parts of fruit sample.

On the other hand, the levels of TPC or TAC determined in all SPE fractions were not significantly differed from the levels of TPC or TAC from the non-fractionated crude extracts of defatted dabai peel (data not shown). This shows that the phenolic compounds in all SPE fractions were fully recovered from SPE. For the antioxidant capacity, TEAC levels of all SPE fractions were 0.7-times lower than the levels determined in the non-fractionated crude extract of defatted dabai peel. It might due to some of the other non-phenolic antioxidants were not recovered from SPE. For defatted dabai pulp-peel, the levels of TPC, TAC and TEAC determined in the non-fractionated crude extract were significantly higher than the SPE fractions. Thus, moderately high recovery of phenolic compounds was assumed for the SPE fractions of defatted dabai pulp. Based on this finding, we postulated that the defatted dabai pulp contains other non-phenolic antioxidants. From our observation, there could have some antioxidant compounds remain absorbed in the CN and C_18_ cartridges. Russo *et al.* [1] also support our observations that some phytochemicals are not able to recover from the elution using certain organic solvents. Therefore, the pool SPE fractions of defatted dabai peel and pulp-peel have lower TAC than the crude extracts.

Surprisingly, C_18_-MeOH fraction from the defatted dabai pulp-peel crude extract had significantly higher levels of TPC, TAC and TEAC than the CN-MeOH fraction. The high TPC determined in the C_18_-MeOH fraction might have some contribution from some unknown phenolic compounds detected in the peaks 12, 13 and 15 (Figure 4). These phenolic compounds have also contributed to the increased antioxidant capacity in the C_18_-MeOH fraction as compared to the CN-MeOH fraction. However, based on the MS result, the relative abundance values of anthocyanins detected in the C_18_-MeOH fraction were lower than in the CN counterpart. Hence C_18_-MeOH fraction of the defatted dabai pulp-peel had significantly higher TAC than all SPE fractions studied (*p* < 0.05). Besides, the levels of phenolic compounds were found to be higher in CN-MeOH fractions than C_18_-MeOH fractions obtained from the defatted dabai peel crude extract, which accessed by both spectrophotometric and chromatographic methods. Spectrophotometric methods may overestimate the TAC determined in the crude extracts of defatted dabai peel and pulp-peel. Khoo *et al.* [22], have mentioned that the UV-Vis spectrophotometric method could overestimate the total antioxidants determined.

In this study, LSD *post-hoc* comparison showed that CN-MeOH fractions of defatted dabai peel had significantly higher TPC and antioxidant capacity than other fractions studied. However, the TAC in CN-MeOH fraction of the defatted dabai peel was lower than its C_18_ counterpart, but not significantly different (*p* > 0.05). As mentioned earlier, spectrophotometric method tend to overestimate the TAC in the defatted dabai peel, especially from C_18_-MeOH and C_18_-water (C_18_-H_2_O) fractions. Based on the MS results, some of the water soluble (polar) phenolic derivatives were poorly retained in the CN-bonded stationary phase. Conversely, anthocyanins were able to retain in the CN-bonded stationary phase. Besides, the anthocyanins detected in the defatted dabai peel were highly soluble in water. Similarly, saponin derivatives that identified in the samples were highly soluble in water, except for the compound that discovered in peak 14. The residual fractions also consisted mainly of saponin derivatives that obtained from both of the defatted dabai peel and pulp-peel crude extracts. 

As we have seen in this study, the anthocyanins that not able to be adsorbed by the stationary phase were considered as waste or residual fraction. In this study, a relatively high amount of anthocyanin (1:10, *w*/*w*) was collected as residual together with some of the phenolic compounds and saponin derivatives. Therefore, before using Sep-Pak^®^ cartridges to fractionate or to concentrate any phenolic compounds from fruit extract, several measures are needed to be considered. Even using multiple cartridges to retain the specific antioxidant compound, there will be a loss of the compound where some of the researchers have overseen it. The use of SPE for fractionation or purification of phenolic compounds has pros and cons [23]. Purification of anthocyanins using Sep-Pak^®^ C_18_ cartridge had been reported by Hong and Wrolstad [24], where the activated C_18_ cartridge was used to adsorb the anthocyanin compounds before eluted by acidic methanol for HPLC analysis. Sep-Pak^®^ C_18_ cartridge had been used to concentrate the anthocyanins isolated using preparative HPLC [25]. Besides CN and C_18_ cartridges, other SPE cartridges are also recommended to be used in fractionation of phenolic compounds in fruits for future studies.

## 3. Experimental

### 3.1. Sample Preparation

Fresh dabai fruits were purchased from three different locations in Sarawak, Malaysia. Dabai kernel was discarded while the pulp and peel of dabai fruit were freeze dried using a bench top freeze dryer (Virtis, New York, NY, USA). The lyophilized samples (peel and pulp-peel) were ground into powder form and defatted using hexane (Fisher Scientific, Loughborough, UK) by soaking overnight. In this study, the sample, pulp-peel refers to dabai pulp without the peel removed. 

### 3.2. Sample Extraction Based on Optimized Method

Extraction of defatted dabai parts (peel and pulp-peel) was based on RSM optimized method, where 2.0 g of the sample was added with 20 mL of 62.25% methanol (Fisher Scientific), and sonicated for 1 min using a Power Sonic 405 Ultrasonicator (Hwashin Technology Co., Seoul, Korea) at 40 kHz to assist extraction. The extraction procedure was previously optimized for obtaining maximum levels of total phenolics and antioxidant capacities (data not shown). All extraction was performed in triplicate. Removal of methanol was done using a rotary evaporator and freeze-dried to remove water. The lyophilized crude extracts were then performed for fractionation of antioxidant compounds using Sep-Pak^®^ cartridges.

### 3.3. Fractionation of Crude Extract by SPE

**Figure 7:**
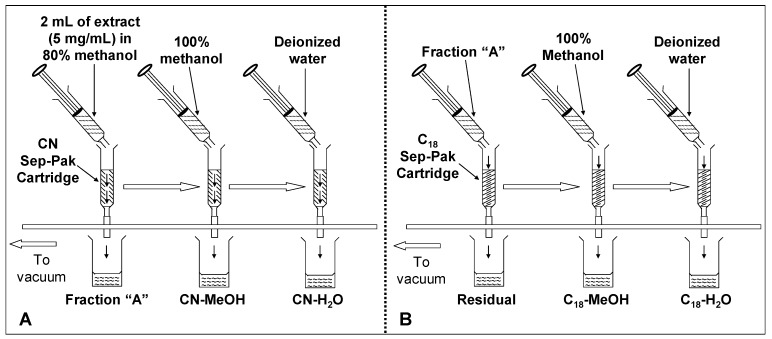
Procedure for fractionation of defatted dabai extracts applying solid phase extraction. Crude extracts of defatted dabai peel and pulp-peel were fractionated based on procedure (**A**), followed by procedure (**B**).

Visprep SPE vacuum manifold (12-ports) (Supelco, Bellefonte, PA, USA) connected to a vacuum pump was used. Sep-Pak^®^ CN and C_18_ cartridges from Merck (Darmstadt, Germany) containing 100 mg adsorbent were used to fractionate the crude extracts of defatted dabai peel and pulp-peel. Fractionation of anthocyanin was performed based on a sequential design using Sep-Pak^®^ CN and C_18_ cartridges. The idea for fractionation of phenolic compounds from the defatted dabai crude extracts was adapted from Russo *et al.* [2], where a Sep-Pak^®^ CN cartridge was connected in “series” with another Sep-Pak^®^ C_18_ cartridge for elution of the compounds. In this study, the elution was performed in stages as shown in Figure 7.

Sep-Pak^®^ cartridges were fixed to the ports of Visprep SPE vacuum manifold. The crude extracts were loaded to the cartridges using 5 mL syringes. First, 2.0 mL of the crude extract (5 mg/mL) was loaded into the activated CN cartridge and the eluent was collected as fraction “A”. Fraction “A” was then loaded to the activated C_18_ cartridge and the eluent was collected as “residual”. The adsorbed or retained phenolic compounds in both of the CN and C_18_ cartridges were eluted by passing 2.0 mL of methanol through the cartridges, followed by 2.0 mL of deionized water. The fractions were collected as CN-H_2_O, CN-MeOH, C_18_-H_2_O and C_18_-MeOH fractions. In this study, equal volumes of the eluent (2 mL each) were collected directly from the cartridges and labeled as the fractions mentioned earlier. A total of five fractions were obtained from each crude extract of either defatted dabai peel or pulp-peel. The fractions were determined for their TPC, TAC and antioxidant capacity (FRAP assay); while potential flavonoids, anthocyanins, and other phytochemicals were detected using LC-MS. Residual fractions were compared with the water and methanolic fractions to ensure all phenolic compounds in the defatted dabai crude extracts have fully recovered from the Sep-Pak^®^ cartridges.

### 3.4. Total Phenolic Content

Total phenolic content (TPC) of the samples were estimated using Folin-Ciocalteu reagent assay [26]. Defatted dabai crude extracts or the SPE fractions (200 μL) were added to 1.5 mL of Folin-Ciocalteau reagent (Sigma, St. Louis, MO, USA) (diluted 10-fold with distilled water). The mixtures were allowed to stand at room temperature for 5 min. Sodium bicarbonate (Sigma) solution (1.5 mL, 60 g/L) was added to the mixtures. After 90 min at room temperature, the absorbance was read at 750 nm using a UV-Vis spectrophotometer (Shimadzu, Kyoto, Japan). TPC was calculated using the regression equations obtained from standard curve of gallic acid (Sigma) (25–150 μg/mL) [9]. The result was expressed as μg gallic acid equivalent (GAE) per mg extract or extract fraction.

### 3.5. Total Anthocyanin Content

Total anthocyanin content (TAC) of the defatted dabai crude extract and the SPE fractions was determined at 535 nm as described by Khoo *et al*. [9]. Briefly, the diluted fraction (2 mL) was measured for the absorption at 535 nm. TAC was calculated based on equation (1):


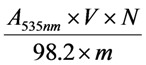
(1)

where TAC is the total anthocyanin content in the crude extract or the SPE fractions (μg/mg), *A_535nm_* is the absorption value measured at 535 nm, *V* is the total volume (mL), *N* is the dilution multiple, 98.2 is the extinction coefficient at 535 nm, and *m* is the mass of crude extract/ SPE fraction.

### 3.6. Trolox Equivalent Antioxidant Capacity

Defatted dabai crude extracts and the SPE fractions were determined for Trolox equivalent antioxidant capacity (TEAC), which based on ferric-reducing antioxidant power (FRAP) assay as described previously [9,27]. Trolox (0.01–0.3 mM) was used for calibration and antioxidant capacity was expressed as mM TEAC per mg extract or extract fraction. 

### 3.7. LC-MS Detection of Potential Antioxidants

LC-MS analyses were performed on a Thermo Quest Finnigan LCQ Deca integrated with a Finnigan Surveyor LC system (Thermo Finnigan, San Jose, CA, USA) and coupled on-line with a Shimadzu QP 8000 API mass spectrometer. PDA and MS data were acquired and processed using Xcalibur software (Thermo Finnigan). The column used was a reversed phase Lichrospher C-18 column (250 × 4 mm, i.d. 5 μm) (Merck) with a guard-cartridge packed with the same stationary phase. 

The chromatographic method was performed based on a method described by Chew *et al.* [10], with some modifications. This method was originally developed by He and Xia [28]. PDA detection was set at 280 nm, with a flow rate of 0.25 mL/min and an injection volume of 20 μL (1 mg/mL). The LC gradient used was: 0–20 min, linear gradient from 0% to 90% B; 20–25 min, 90% B isocratic; 25–30 min, linear gradient from 90% to 0% B. MS conditions were nebulizer gas flow of 4.5 L/min, probe voltage of 4.5 kV, curved desolvation line (CDL) voltage of 130.0 V, CDL temperature of 230 °C, deflector voltage at 45 and 60 V, and scan speed of 2,000 amu/s [29]. Data were acquired based on positive and negative ionization mode from *m/z* 100 to 1,000 in 10 s. The present study has a high repeatability as the average relative standard deviation was lower than 10%.

### 3.8. Statistical Analysis

TPC, TAC and antioxidant capacity of the defatted dabai peel and pulp-peel crude extracts and their SPE fractions were expressed as means ± standard deviations. Significant differences between the means of different samples were determined by one-way analysis of variance and further compared using LSD post-hoc test. Statistical software used was Minitab version 15 (Minitab Inc., State College, PA, USA). The significant difference was set at *p* < 0.05.

## 4. Conclusions

Extraction and fractionation of phenolic antioxidants based on SPE enable separation of specific phenolic compounds, such as flavonoids and anthocyanins. CN and C_18_ bonded-phases cartridges used have provided new knowledge on the type of phenolic compounds fractionated from defatted dabai samples. Hesperetin glucoside was not detected in the defatted dabai pulp, while two unknown phytochemicals were not found in the peel of the defatted dabai. A high TPC was determined in CN-MeOH fractions from the defatted dabai peel as compared to the other SPE fractions. Some unknown phytochemicals were not detected in the methanolic fractions of defatted dabai pulp-peel; however, these compounds were spotted in the water fractions, and vice versa. Higher level of anthocyanins was retained by the CN bonded-phase than the C_18_ bonded-phased, while water had higher ability to elute anthocyanin glucosides from the defatted dabai peel compared to methanol. Applying SPE for fractionation of defatted dabai crude extract, a different profile of phenolic compounds was observed. The used of SPE in extraction and fractionation of phenolic compounds is not advisable as a lost of phenolic compounds was observed in this study. The best way to determine the phenolic compounds or antioxidant capacity of any fruits of interest is to analyze the crude extract instead to fractionate or purify it. 
